# An (*E,E*)‐α‐farnesene synthase gene of soybean has a role in defence against nematodes and is involved in synthesizing insect‐induced volatiles

**DOI:** 10.1111/pbi.12649

**Published:** 2016-11-17

**Authors:** Jingyu Lin, Dan Wang, Xinlu Chen, Tobias G. Köllner, Mitra Mazarei, Hong Guo, Vincent R. Pantalone, Prakash Arelli, Charles Neal Stewart, Ningning Wang, Feng Chen

**Affiliations:** ^1^ Department of Plant SciencesUniversity of TennesseeKnoxvilleTNUSA; ^2^ Department of Plant Biology and EcologyCollege of Life SciencesNankai UniversityTianjinChina; ^3^ Department of BiochemistryMax Planck Institute for Chemical EcologyJenaGermany; ^4^ Department of BiochemistryCellular and Molecular BiologyUniversity of TennesseeKnoxvilleTNUSA; ^5^ Crop Genetics Research UnitUSDA‐ARSJacksonTNUSA

**Keywords:** sesquiterpene synthase, volatile, transgenic hairy roots

## Abstract

Plant terpene synthase genes (*TPSs*) have roles in diverse biological processes. Here, we report the functional characterization of one member of the soybean *TPS* gene family, which was designated *GmAFS*. Recombinant GmAFS produced in *Escherichia coli* catalysed the formation of a sesquiterpene (*E,E*)‐α‐farnesene. *GmAFS* is closely related to (*E,E*)‐α‐farnesene synthase gene from apple, both phylogenetically and structurally. *GmAFS* was further investigated for its biological role in defence against nematodes and insects. Soybean cyst nematode (SCN) is the most important pathogen of soybean. The expression of *GmAFS* in a SCN‐resistant soybean was significantly induced by SCN infection compared with the control, whereas its expression in a SCN‐susceptible soybean was not changed by SCN infection. Transgenic hairy roots overexpressing *GmAFS* under the control of the CaMV 35S promoter were generated in an SCN‐susceptible soybean line. The transgenic lines showed significantly higher resistance to SCN, which indicates that *GmAFS* contributes to the resistance of soybean to SCN. In soybean leaves, the expression of *GmAFS* was found to be induced by *Tetranychus urticae* (two‐spotted spider mites). Exogenous application of methyl jasmonate to soybean plants also induced the expression of *GmAFS* in leaves. Using headspace collection combined with gas chromatography–mass spectrometry analysis, soybean plants that were infested with *T. urticae* were shown to emit a mixture of volatiles with (*E,E*)‐α‐farnesene as one of the most abundant constituents. In summary, this study showed that *GmAFS* has defence roles in both below‐ground and above‐ground organs of soybean against nematodes and insects, respectively.

## Introduction

Soybean is a crop of global importance. Its yield can be significantly reduced due to diseases caused by microbial pathogens and infestation by herbivorous insects (Hartman *et al*., 2011). The approaches to managing biotic agents of soybean plants, similar to other major crops, include sound cultural practices, application of synthetic pesticides and deployment of resistant cultivars (Oerke, 2006). The development of disease/insect‐resistant soybean may be assisted by the mechanistic elucidation of plant natural defences, especially the isolation of defence genes. The production of secondary metabolites is one strategy of plant natural defences against pathogens and insects (Bennett and Wallsgrove, 1994; Zhao *et al*., 2013). The most structurally diverse group of plant secondary metabolites is terpenoids, which have diverse roles in the interactions of plants with the environment, including serving as defences against pathogens and insects (Gershenzon and Dudareva, 2007). The soybean genome has been fully sequenced (Schmutz *et al*., 2010). This valuable resource is expected to facilitate the identification of candidate genes involved in the biosynthesis of terpenoids, especially of those that have roles in natural defences of soybean plants.

Terpene synthases (TPSs) are key enzymes for terpene biosynthesis. They catalyse the formation of terpenes from isoprenyl diphosphate substrates of various chain lengths (Degenhardt *et al*., 2009). In flowering plants, *TPSs* form a mid‐sized gene family in each species (Chen *et al*., 2011). Over the past 10 years, we have been engaged in functional characterization of the *TPS* gene family in natural defences in several crop plants, including rice (Yuan *et al*., 2008), sorghum (Zhuang *et al*., 2012) and poplar trees (Danner *et al*., 2011). We have also embarked on a project to study the *TPS* family of soybean. A recent study (Liu *et al*., 2014) showed that the soybean *TPS* family (*GmTPSs*) consists of more than 20 members. The expression of 21 *GmTPS* genes was examined in different soybean tissues. While many genes were found to be expressed in primarily reproductive organs, twelve *GmTPS* genes also showed different expression patterns in response to mechanical wounding (Liu *et al*., 2014). *GmTPS3* was determined to encode geraniol synthase, and transgenic tobaccos overexpressing *GmTPS3* showed increased resistance to cotton leaf worms (Liu *et al*., 2014).

In this study, we report the functional characterization of *GmTPS21*, which we designated *GmAFS* (*G. max* α‐farnesene synthase). *GmAFS* was selected for this investigation because it was identified as one of the candidate defence genes against soybean cyst nematodes (SCNs) in our previous study (Mazarei *et al*., 2011). SCN is the most important pathogen of the soybean crop (Koenning and Wrather, 2010). Thus, it has been highly desired to identify and isolate soybean defence genes for genetic improvement of soybean for enhanced resistance against SCN. There were three objectives in this study. The first objective was to determine the biochemical function of the protein encoded by *GmAFS*. Terpene synthases can be categorized into monoterpene synthases, sesquiterpene synthases, and diterpene synthases, depending on the products they form (Chen *et al*., 2011). We used *in vitro* biochemistry to determine the specific biochemical activity of GmAFS. The second objective was to determine whether *GmAFS* indeed has a role in SCN resistance. For this objective, transgenic hairy roots overexpressing *GmAFS* were produced and assayed for SCN resistance. The third objective was to examine whether *GmAFS* has roles in soybean defence against other pests. In many plant systems, insect herbivory can induce the biosynthesis and emission of volatile terpenoids (Shrivastava *et al*., 2010). For the third objective, we specifically examined whether *GmAFS* has a role in soybean defence against insects in above‐ground tissues.

## Results

### Expression of *GmAFS* is induced by SCN infection in SCN‐resistant soybean

**Figure 1:**
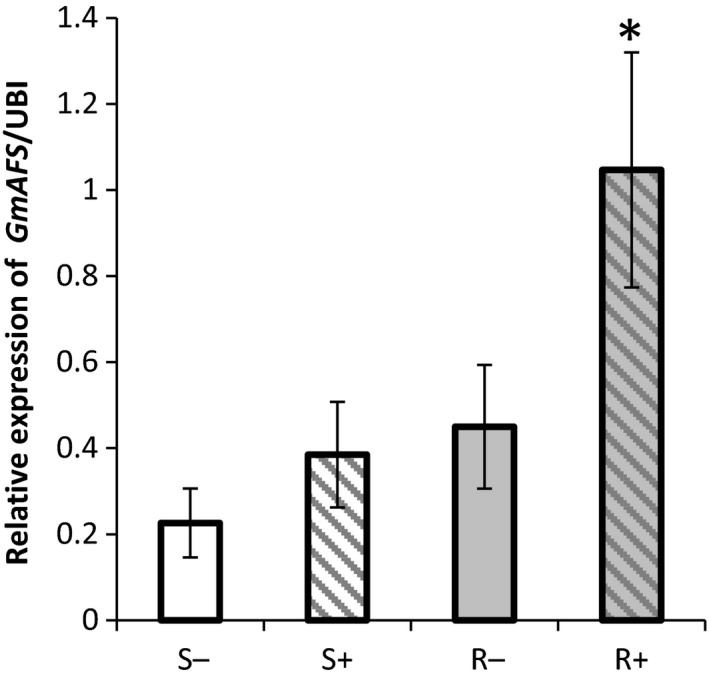
Expression of *GmAFS* gene in the SCN‐infected (+) and noninfected control (−) roots of the SCN‐resistant (R) and SCN‐susceptible (S) soybean lines using quantitative RT‐PCR. The expression of *GmAFS* was examined in the root tissues from TN02‐226 (R) and TN02‐275 (S) soybean breeding lines with (+)/without (−) the treatment of SCN HG type 1.2.5.7 (race 2). The PCR products for soybean ubiquitin‐3 (*GmUBI‐3*) were used to judge equality of concentration of cDNA templates in different samples. Bars represent mean values of three biological replicates with standard error. Bars with asterisks are significantly different at *P *< 0.05 as tested by Fisher's least significant difference.

In our previous GeneChip analysis, the expression of *GmAFS* corresponding to Gma.625.1.S1_at was shown to be significantly induced by SCN infection in the SCN‐resistant soybean TN02‐226, whereas gene expression was unchanged in SCN‐infected susceptible (TN02‐275) plants (Mazarei *et al*., 2011; see Supplemental Table 2 therein). As it is possible that false‐positive results could occur in microarray experiments from cross‐hybridization (Dai *et al*., 2002), quantitative reverse‐transcription PCR (qRT‐PCR) experiments were performed. First, root tissues were collected from the SCN‐resistant soybean line TN02‐226 and the SCN‐susceptible soybean line TN02‐275 with (3 days post‐SCN inoculation) and without SCN infection. These samples were subject to qRT‐PCR analysis for *GmAFS*. No significant difference was observed in the expression of *GmAFS* in the SCN‐susceptible soybean with or without SCN infection. In contrast, the expression of *GmAFS* in the SCN‐resistant soybean was significantly increased (about 2.5‐fold) by SCN infection in comparison with that of the control roots without SCN infection (Figure 1).

### Evolutionary relatedness of GmAFS with other terpene synthases

**Figure 2:**
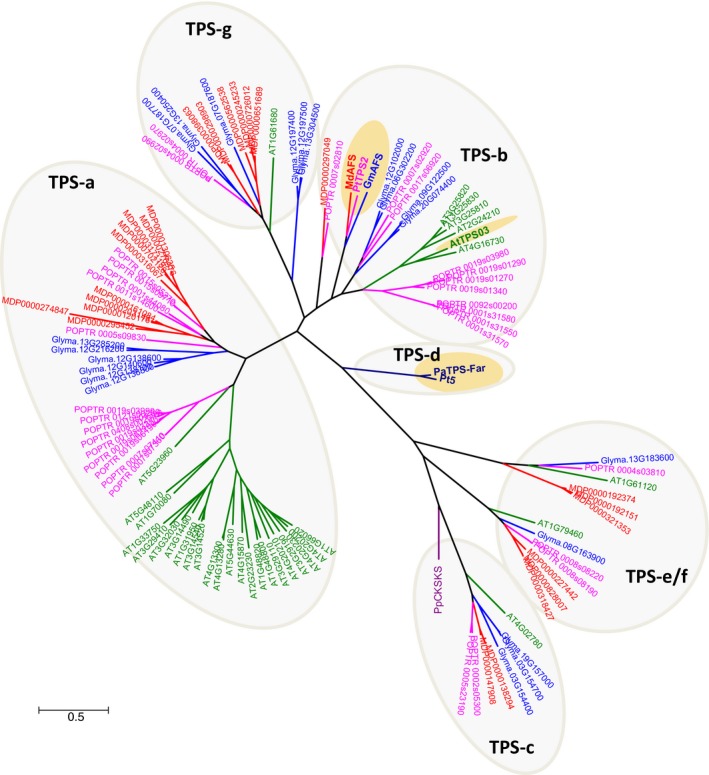
Phylogenetic tree of terpene synthases (TPSs) from soybean (blue), apple (red), poplar (pink), *Arabidopsis* (green) and representative ones from gymnosperm. PpCPS/KS is a diterpene synthase from the moss *Physcomitrella patens*; it resembles ancestral plant terpene synthases. GmAFS is the terpene synthase gene from soybean which was investigated in this study. MdAFS (GenBank accession AAO22848.2) and PtTPS2 (GenBank accession AEI52902) are known (*E,E*)‐α‐farnesene synthases from apple and poplar, respectively. AtTPS3 from Arabidopsis also encodes (*E,E*)‐α‐farnesene synthase (Fäldt *et al*., 2003). PaTPS‐far (GenBank accession AAS47697) and Pt5 (GenBank Accession AAO61226) are (*E,E*)‐α‐farnesene synthase from *Picea abies* and *Pinus taeda*, respectively. TPS‐a, b, c, d, e/g and g depict subfamilies. All known (*E,E*)‐α‐farnesenes are highlighted in yellow.

**Figure 3:**
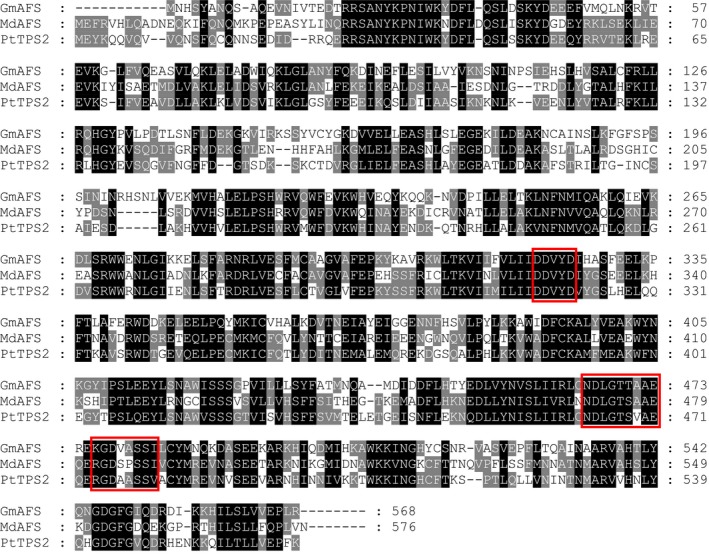
Protein sequence alignment of GmAFS with (*E,E*)‐α‐farnesene synthases from apple (MdAFS) and poplar (PtTPS2). Three conserved motifs among GmAFS, MdAFS1 and PtTPS2 are boxed: the ‘DDxxD’ motif, the ‘NSD/DTE’ motif and the ‘H‐α1 loop’.

With the confirmation of *GmAFS* expression in relation to SCN infestation (Figure 1), our next objective was to determine the biochemical function of the protein encoded by *GmAFS*. To this end, we first performed phylogenetic analysis to determine the evolutionary relatedness of GmAFS with other TPSs including some with known functions. From the latest version of the annotated soybean genome, 22 putative full‐length TPS genes including *GmAFS* (*Glyma.13G321100*) were identified. Phylogenetic trees were reconstructed using all TPSs from four sequenced dicot plants: soybean, Arabidopsis, apple and poplar. From this analysis, the soybean TPSs were determined to belong to five subfamilies: a, b, c, e/g and g (Figure 2). GmAFS is a member of the TPS‐b subfamily. Most members of the TPS‐b subfamily encode monoterpene synthase (Chen *et al*., 2011), except apple (*E,E*)‐α‐farnesene synthase (MdAFS) (Green *et al*., 2007) and poplar (*E,E*)‐α‐farnesene synthase (PtTPS2) (Danner *et al*., 2011), which are sesquiterpene synthases. GmAFS clustered together with MdAFS and PtTPS2 (Figure 2) and showed 53% and 51% sequence identity to MdAFS and PtTPS2, respectively. Besides sharing high overall sequence similarity, GmAFS, MdAFS and PtTPS2 exhibit conserved structural features. These include the aspartate‐rich DDxxD motif, NSD/DTE motif, and H‐α1 loop (Figure 3). The H‐α1 loop of apple MdAFS has been previously demonstrated to function in the binding of the metal ion K^+^ (Green *et al*., 2009). Evolutionary relatedness, sequence homology and conserved structural features suggested that *GmAFS* encodes (*E,E*)‐α‐farnesene synthase.

### *GmAFS* encodes a sesquiterpene synthase producing (*E,E*)‐α‐farnesene

**Figure 4:**
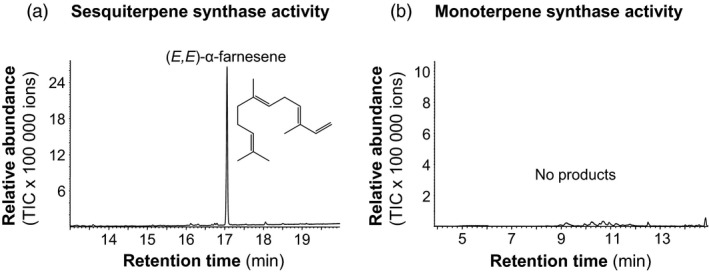
GC–MS total ion chromatograms of reaction products from a terpene synthase assay of *E. coli*‐expressed GmAFS. (a) The terpene synthase assay of *E. coli*‐expressed recombinant GmAFS using farnesyl diphosphate (FPP) as substrate showing the sesquiterpene synthase activity. (b) The terpene synthase assay of *E. coli* expressed GmAFS using geranyl diphosphate (GPP) as substrate showing no monoterpene synthase activity.

Under the standard assay conditions (Zhuang *et al*., 2012), GmAFS did not show activity with either geranyl diphosphate or farnesyl diphosphate. As it has been demonstrated that the (*E,E*)‐α‐farnesene synthases from apple and poplar need K^+^ in addition to magnesium for catalytic activity (Danner *et al*., 2011; Green *et al*., 2009), we also performed assays containing K^+^. In the presence of K^+^, GmAFS converted farnesyl diphosphate into (*E,E*)‐α‐farnesene (Figure 4a). However, GmAFS was not able to convert geranyl diphosphate into monoterpenes under the same conditions (Figure 4b).

### Structural feature of the GmAFS model and comparison with that of MdAFS

**Figure 5:**
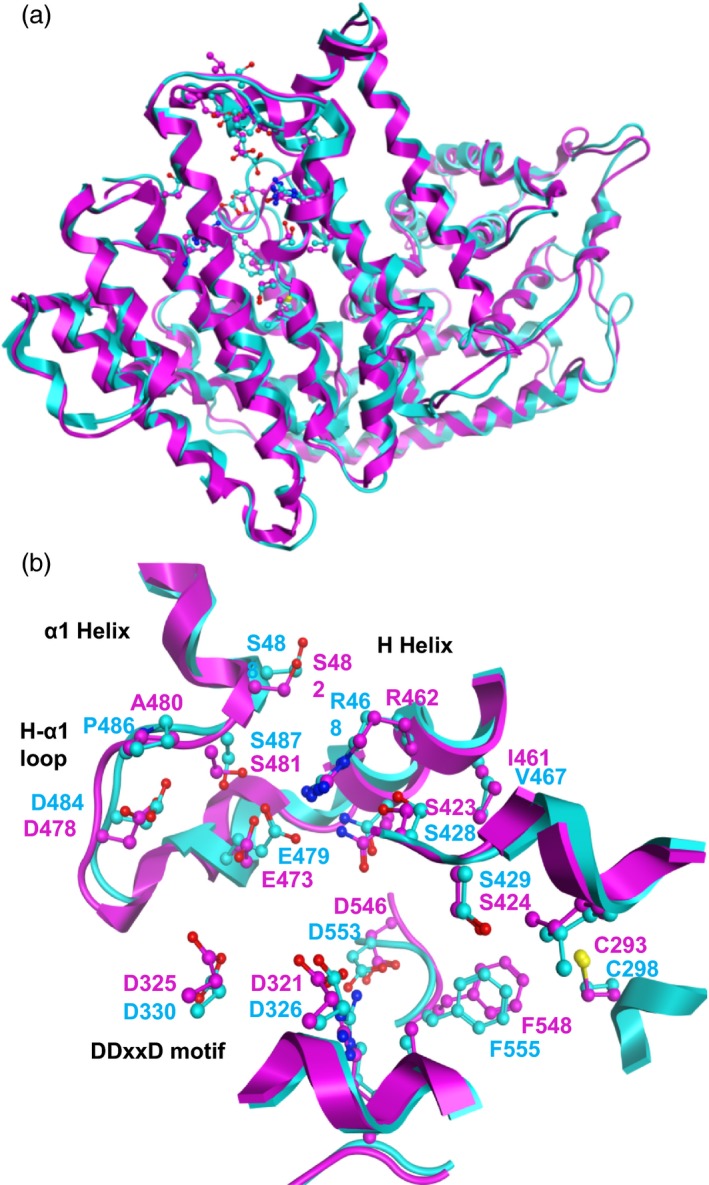
Structural analysis of GmAFS. (a) Supposition of the homology models for GmAFS (magenta) and MdAFS (cyan). The two models can be superposed well with a RMSD deviation of 1.5 Å. The active site residues are shown in ball and stick. (b) The active site structures and residues from the GmAFS and MdAFS models. H‐α1 loop, DDxxD motif and H helix are also shown.

With the experimental confirmation that *GmAFS* encodes (*E,E*)‐α‐farnesene synthase (Figure 4), next, we compared the three‐dimensional structures and the active sites of GmAFS and MdAFS. Homology models of GmAFS and MdAFS were generated by use of the structure of tobacco 5‐*epi*‐aristolochene synthase (PDB ID: 5EAT) as template (Facchini and Chappell, 1992). The two models superpose well with a RMSD deviation of only 1.5 Å (Figure 5a). The models for GmAFS and MdAFS obtained with the X‐ray structure of (+)‐bornyl diphosphate synthase (1N20), a monoterpene synthase (Whittington *et al*., 2002), as the template are also similar. The active site residues for GmAFS and MdAFS were found to be conserved. These residues as well as structural motifs (e.g., H‐α1 loop, DDxxD motif and H helix) in GmAFS and MdAFS were well aligned (Figure 5b). It is of interest to note that the H‐α1 loops from the two models superpose well, even though the residue corresponding to P486 in MdAFS is Ala (A480) in GmAFS.

### Overexpressing *GmAFS* in transgenic hairy roots led to enhanced resistance to soybean cyst nematode

**Figure 6:**
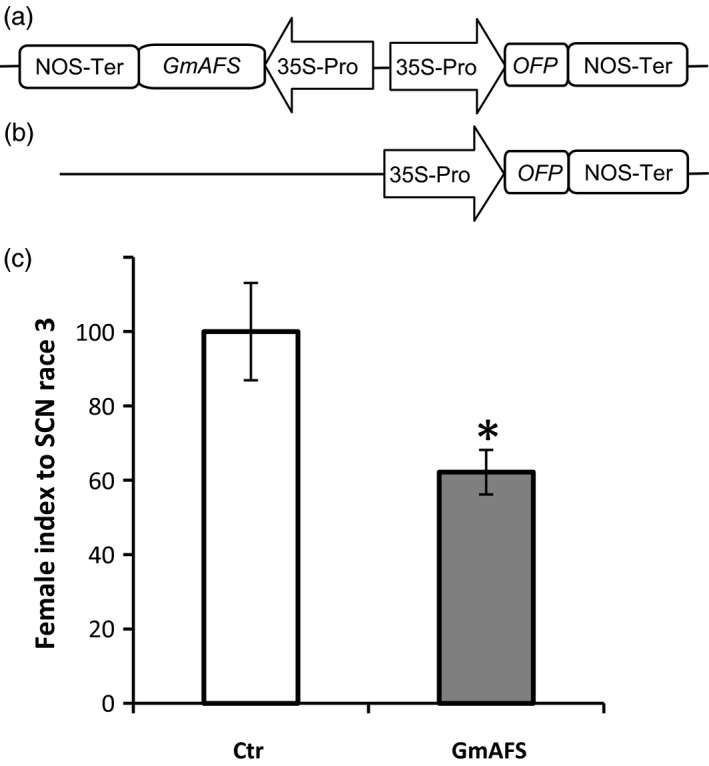
Susceptibility of transgenic soybean hairy roots with overexpression of *GmAFS* to SCN race 3. (a) Schematic representation of the construct used for transgenic soybean line overexpressing *GmAFS* and an orange fluorescent protein (OFP) reporter gene. ‘35S‐Pro’ and ‘NOS‐ter’ represent the CaMV 35S promoter and the NOS terminator, respectively. (b) Schematic representation of the construct used for control line containing only *OFP* reporter gene. (c) Ctr stands for the soybean hairy root transformed with the vector containing an orange florescence protein gene. GmAFS stands for the soybean hairy root transformed with the construct overexpressing *GmAFS*. Williams 82 soybean was the variety used for generating these two types of hairy roots. Bars represent mean values (*n* = 20) of the female index with standard error. Bars with asterisks are significantly different at *P *< 0.05 as tested by Fisher's least significant difference.

The expression pattern of *GmAFS* (Figure 1) strongly suggested that *GmAFS* may have a role in soybean defence against SCN. To test this hypothesis, we chose to use transgenic hairy root system, which has been proved in our previous studies to be a reliable system for evaluating candidate SCN‐resistant genes (Lin *et al*., 2013). A SCN‐susceptible line of soybean (Williams 82) was used to produce transgenic hairy roots overexpressing *GmAFS* under control of CaMV35S promoter. For fast screening, transgenic hairy roots were generated to coexpress *GmAFS* and an orange florescence protein (OFP) reporter gene (Figure 6a) in the same cassette. As a negative control, transgenic hairy roots were also produced with a binary vector containing only the ORP reporter gene under the control of CaMV35S promoter (Figure 6b). There was no significant difference on generating the hairy roots between the *GmAFS*‐overexpressing line and the vector control line.

The mean number of adult females and cysts for the control line was about 17.0, whereas the mean number of adult females and cysts for *GmAFS*‐overexpressing transgenic hairy roots was about 10.6. Significantly fewer cysts were observed in transgenic soybean hairy root overexpressing *GmAFS* than that from control transgenic hairy roots. The female index of transgenic hairy roots overexpressing *GmAFS* (approximately 60) was significantly lower than that of the control (artificially set to 100) (Figure 6c), which means that the transgenic soybean showed 40% decrease in susceptibility to SCN.

### The expression of *GmAFS* in soybean leaves can be induced by herbivory and methyl jasmonate

**Figure 7:**
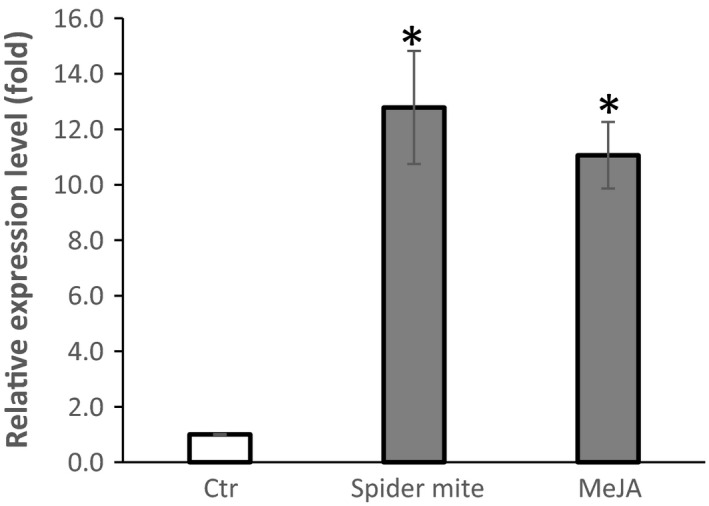
Expression of *GmAFS* gene in of the leaves of the Williams 82 soybean plants that were infested by *T. urticae* (spider mite), treated by methyl jasmonate (MeJA) and control plants (Ctr) using quantitative RT‐PCR. The PCR products for soybean ubiquitin‐3 (*GmUBI‐3*) were used to judge equality of concentration of cDNA templates in different samples. Bars represent mean values of three biological replicates standard error. Bars with asterisks are significantly different at *P *< 0.05 as tested by Fisher's least significant difference.

Some terpene synthase genes are known to be expressed in multiple tissues and can be induced by multiple stresses (Fäldt *et al*., 2003). To understand whether *GmAFS* has roles in other biological processes other than defence again SCN, we examined the expression of *GmAFS* in leaves especially under stress conditions using qRT‐PCR. The expression of *GmAFS* in leaves infested with *Tetranychus urticae* (two‐spotted spider mite) was found to be 12‐fold higher than its expression in control soybean leaves without *T. urticae* infestation (Figure 7). The jasmonate signalling pathway is essential for regulating plant defence responses to insect herbivory (War *et al*., 2012). To understand whether this pathway is also associated in regulating the expression of *GmAFS*, soybean plants were treated with methyl jasmonate and leaves were collected for gene expression analysis. The expression of *GmAFS* in the methyl jasmonate‐treated plants was 11‐fold higher than its expression in the control plants (Figure 7).

### (*E,E*)‐α‐farnesene was one of the major volatile compounds emitted from soybean plants infested with *T. urticae*

**Figure 8:**
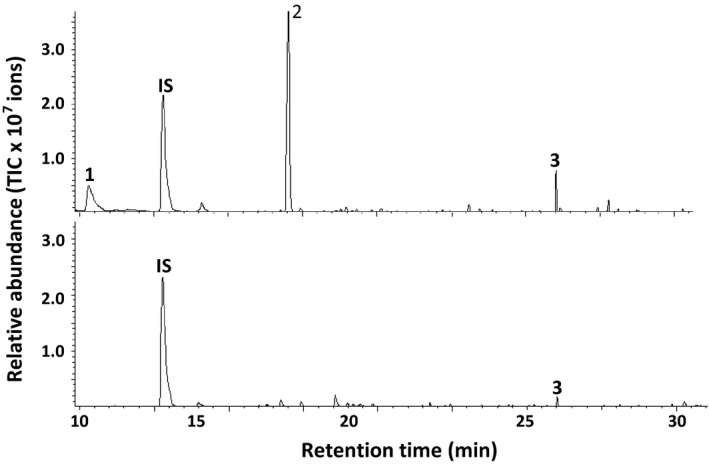
Volatiles emitted from Williams 82 soybean plants infested with two‐spotted spider mites (*Tetranychus urticae*). The untreated Williams 82 soybean plants were analysed as a control. The upper panel shows a GC chromatogram of the volatiles from *T. urticae*‐infested plants, and the lower panel shows a GC chromatogram from control plants. IS represents the internal standard. 1, *Z*‐3‐hexenyl acetate; 2, methyl salicylate; 3, (*E,E*)‐α‐farnesene. The volatile profiling experiment was repeated three times with similar results.

Gene expression analysis of *GmAFS* in soybean leaves showed that this gene is induced by herbivory (Figure 8). To determine whether (*E,E*)‐α‐farnesene, the product of GmAFS, is released as a volatile compound from *T. urticae*‐infested soybean plants, we performed dynamic headspace collection coupled with gas chromatography–mass spectrometry analysis. (*E,E*)‐α‐farnesene was among the major volatile compounds detected from the *T. urticae*‐infested soybean plants. Other major compounds include *Z*‐3‐hexenyl acetate and methyl salicylate (Figure 8). Untreated control soybean plants were also analysed. While (*E,E*)‐α‐farnesene was also detected, its amount was lower than that from the *T. urticae*‐infested soybean plants (Figure 8).

## Discussion

### GmAFS provides novel information about the evolution of terpene synthases

Terpene synthase genes form subfamilies with individual subfamilies usually associated with specific biochemical functions as monoterpene synthases, sesquiterpene synthases or diterpene synthases (Chen *et al*., 2011). GmAFS belongs to the TPS‐b subfamily, members of which generally function as monoterpene synthases. It is therefore somehow surprising to observe that GmAFS functions as a sesquiterpene synthase (Figure 4). Further phylogenetic analysis provided new insight into the evolution of the TPS‐b subfamily in general and the (*E,E*)‐α‐farnesene synthases clade in particular. The (*E,E*)‐α‐farnesene synthase genes from soybean and apple are apparent orthologs, implying that their immediate ancestor gene evolved in the common ancestor of Fabidae. *GmAFS* and *MdAFS* being a pair of orthologs are also being supported by the high structural similarities of the two proteins they encode (Figure 5). There is conflicting evidence in regard to whether this gene evolved in the common ancestor of Rosid. The clustering of the poplar (*E,E*)‐α‐farnesene synthase gene with the apple and soybean (*E,E*)‐α‐farnesene synthase genes supports this hypothesis. However, the (*E,E*)‐α‐farnesene synthase gene from Arabidopsis (AtTPS03) does not support this hypothesis. In addition to the phylogenetic evidence presented in this paper (Figure 2), the Arabidopsis (*E,E*)‐α‐farnesene synthase behaves differently at the biochemical level. While GmAFS, MdAFS and PtTPS2 all use K^+^ and Mg^2+^ as cofactors, AtTPS03 uses only Mg^2+^ as a factor like most TPSs (Huang *et al*., 2010). It is certainly possible that the orthologous (*E,E*)‐α‐farnesene synthase gene was lost in Arabidopsis. The analysis of the putative orthologs of this gene in other species of Rosid will provide evidence for testing this hypothesis. Prior to this study, several (*E,E*)‐α‐farnesene synthase genes have been isolated from gymnosperms (Phillips *et al*., 2003), which belong to the TPS‐d subfamily (Figure 2). Together, these results suggest that (*E,E*)‐α‐farnesene synthase genes have evolved multiple times in seed plants.

### GmAFS has a role in soybean defence against SCN

The induced expression of *GmAFS* in SCN‐resistant soybean suggested that GmAFS has a role in soybean defence against SCN (Figure 1). This hypothesis was supported with the overexpression of *GmAFS* in transgenic hairy roots of a soybean variety that is SCN susceptible (Figure 6). Interestingly, the expression pattern of *GmAFS* is highly similar to that of soybean salicylic acid methyltransferase gene (*GmSAMT1*) (Lin *et al*., 2013). The expression of *GmSAMT1* in a SCN‐susceptible line was not significantly changed with SCN infection. In contrast, its expression was significantly induced by SCN infection in a SCN‐resistant line. Similarly, overexpression of *GmSAMT1* in a SCN‐susceptible line also led to enhanced resistance to SCN (Lin *et al*., 2013). The enhanced resistance of *GmSAMT1*‐overexpressors to SCN was attributed to the changes in the salicylic acid signalling pathway (Lin *et al*., 2013). While the defence mechanism conferred by GmAFS is unclear, it is tempting to speculate that its product (*E,E*)‐α‐farnesene has nematicidal activity. A number of terpenoids have been demonstrated to be nematicidal (Ntalli *et al*., 2010; Oka *et al*., 2000). It will be interesting to determine whether the defence rendered by *GmAFS* is indeed due to the toxicity of (*E,E*)‐α‐farnesene or other mechanisms. It will also be interesting to determine whether *GmAFS*,* GmSAMT1* and other SCN‐resistant genes work concertedly to achieve SCN resistance.

### *GmAFS* may be involved in indirect defence against insects

In addition to defence against SCN, *GmAFS* is suggested to have a role in soybean defence against herbivorous insects. When infested by soybean aphids, soybean plants emitted a mixture of volatile compounds including (*E,E*)‐α‐farnesene (Moraes *et al*., 2005). Aphid‐induced volatiles from soybean plants were shown to attract soybean aphid's natural enemies such as Syrphidae (Diptera), Chrysopidae (Neuroptera), and green lacewings (Mallinger *et al*., 2011). Volatile (*E,E*)‐α‐farnesene has been shown to be active signal in attracting natural enemies. Laboratory results showed that α‐farnesene was attractive to parasitic wasps including *Aphidius ervi*,* Coleomegilla maculate* and *Chrysoperla carnea* (Du *et al*., 1998; Zhu *et al*., 1999). James (2005) also reported the parasitic mymarid wasp, *Anagrus daanei*, was attracted to farnesene in a field study. In the current study, *T. urticae* infestation induced the expression of *GmAFS* (Figure 7) and elevated emission of (*E,E*)‐α‐farnesene (Figure 8), suggesting that (*E,E*)‐α‐farnesene may have a role in attracting the natural enemies of *T. urticae* as well. In fact, (*E,E*)‐α‐farnesene is a common compound of herbivore‐induced plant volatile blends from some plants such as bean, pear, apple and poplar (Boeve *et al*., 1996; Danner *et al*., 2011; Du *et al*., 1998; Scutareanu *et al*., 2003). Therefore, (*E,E*)‐α‐farnesene might function in indirect defence pertaining to many insect pests for many plants, including soybean.

### Other possible roles of *GmAFS* and its use for genetic improvement of soybean

In addition to its roles in defence against SCN and indirect defence against insects, GmAFS may also have other functions. For instance, the product of GmAFS may provide defence against bacterial pathogens, fungal pathogens and viruses. In a previous study, root‐emitted sesquiterpene caryophyllene was shown to attract beneficial nematodes for defence against insects (Rasmann *et al*., 2005). It will be interesting to determine whether GmAFS‐produced (*E,E*)‐α‐farnesene has a similar function. *GmAFS* (*GmTPS21*) was found to be highly expressed in stem and mature flowers and its expression increased nearly 25‐fold after 2 h of wounding (Liu *et al*., 2014). Besides such functional studies, *GmAFS* presents a useful genetic improvement of soybean for enhanced defence against multiple biotic agents. We are in the process of producing transgenic soybean overexpressing *GmAFS*. Once produced, we will test these lines for defence function against individual pathogens and insects as well as the agronomic performance of the transgenic soybean in the field.

## Experimental procedures

### Plants, insects and plant treatments

Three soybean (*Glycine max*) lines were used in this study. These included ‘Williams 82’ and two genetically related breeding lines: TN02‐226 and TN02‐275, which are resistant and susceptible, respectively, to soybean cyst nematode (SCN) HG type 1.2.5.7 (race 2). TN02‐226 and TN02‐275 were used in our prior gene profiling to identify candidate SCN‐resistant genes (Mazarei *et al*., 2011). Williams 82 soybean was used as the plant materials for SCN HG type 0 (race 3) bioassay and gene expression examination under two‐spotted spider mites (*Tetranychus urticae*) and methyl jasmonate treatment. Chlorine gas‐sterilized soybean seeds were germinated on autoclaved filter paper moistened with sterile distilled water. Three soybean seedlings were grown in a single pot under 150–200 μmol m^−2^ s^−1^ irradiance 12‐h light/12‐h dark cycle at 28 °C/25 °C for 21 days. For the methyl jasmonate treatment, 3‐week‐old Williams 82 seedlings were irrigated with 25 mL of 5 mM methyl jasmonate. After 24 h, leaves were harvested for gene expression analysis. For the insect treatment, 3‐week‐old Williams 82 soybean seedlings were transferred to the glasshouse for treatment with *T. urticae*. A total of 200 spider mites were used to infest one soybean plant. After 3 days of infestation, soybean plants were subjected to volatile profiling. After volatile profiling, leaves were harvested for gene expression analysis.

### Database search and sequence analysis

TPS genes from soybean and apple were identified by analysing their respective genome sequences housed at Phytozome v9.1 (http://www.phytozome.net) using Blast search. The TPS genes from Arabidopsis and poplar were from a previous dataset (Chen *et al*., 2011). Multiple protein sequence alignment was performed using ClustalX 2.1 (Larkin *et al*., 2007). A maximum‐likelihood tree was constructed using MEGA 6.0 (Tamura *et al*., 2013) with the Jones–Taylor–Thornton (JTT) model and bootstrapping of 1000 replicates.

### Isolation of full‐length cDNA of *GmAFS*

A full‐length cDNA of *GmAFS* was isolated via RT‐PCR from soybean roots infested by SCN. Total RNA was extracted from root tissues using the RNeasy Plant Mini Kit (Qiagen, Valencia, CA), and DNA contamination was removed with DNase treatment following the manufacturers' instructions. Then purified, total RNA was reverse‐transcribed into first‐strand cDNA in a 15 μL reaction volume using the First‐Strand cDNA Synthesis Kit (GE Healthcare, Piscataway, NJ) as previously described (Chen *et al*., 2003). The following primers were designed for cloning and semiquantitative RT‐PCR as follows: *GmAFS*‐F: 5′‐ATGAATCACTCATACGCGAATCAATC‐3′ and *GmAFS*‐R: 5′‐CTATCTAAGGGGTTCAACAACCAGTG‐3′. The PCR program used to amplify the target genes was performed as follows: 94 °C for 2 min followed by 35 cycles at 94 °C for 30 s, 56 °C for 30 s and 72 °C for 1 min 50 s, and a final extension at 72 °C for 10 min. PCR products were cloned into vector pEXP5/CT‐TOPO and fully sequenced.

### *Escherichia coli* expression of GmAFS and terpene synthase enzyme assay

The assays were conducted in standard assay conditions, as previously reported (Zhuang *et al*., 2012). To study the biochemical function of GmAFS, the above‐mentioned protein expression vector pEXP5/CT‐TOPO harbouring *GmAFS* was transformed into the *E. coli* strain BL21 (DE3) CodonPlus (Stratagene, La Jolla, CA). Fifty millilitres of liquid cultures of the bacteria harbouring the expression constructs were grown at 37 °C to an OD600 of 0.6. Isopropyl β‐D‐1‐thiogalactopyranoside (IPTG) with the final concentration of 500 μm was added to the culture for induction, and the cells were kept cultured for 20 h at 18 °C. Then, the cells were collected by centrifugation and disrupted by a 4 × 30 sec sonication treatment in chilled extraction buffer (50 mM Mopso, pH 7.0, with 5 mM MgCl_2_, 5 mM sodium ascorbate, 0.5 mM PMSF, 5 mM dithiothreitol and 10% (v/v) glycerol). The cell fragments were removed by centrifugation at 14 000 ***g***, and the supernatant was desalted into assay buffer (10 mM Mopso, pH 7.0, 1 mM dithiothreitol, 10% (v/v) glycerol) by passage through an Econopac 10DG column (Bio‐Rad, Hercules, CA).

The enzyme assays for recombinant GmAFS were performed at 30 °C for 1 h, using 50 μL of the crude enzyme and 50 μL assay buffer with 10 μm substrate (geranyl diphosphate and farnesyl diphosphate, respectively), 10 mM MgCl_2_, 0.05 mM MnCl_2_, 50 mM KCl, 0.2 mM NaWO_4_ and 0.1 mM NaF in a Teflon‐sealed, screw‐capped 1 mL GC glass vial. A solid‐phase microextraction (SPME) fibre consisting of 100 μm polydimethylsiloxane (Supelco, Bellefonte, PA) was placed in the headspace of the vial for 10 min for collecting the volatile. For analysis of the absorbed reaction products, the SPME fibre was inserted directly into the injector of the gas chromatograph. GC‐MS analysis and product identification were performed as described below.

### Homology models

Homology models were built for both GmAFS and apple (*E,E*)‐α‐farnesene (MdAFS) based on the X‐ray structure of Tobacco 5‐*epi*‐Aristolochene Synthase (PDB ID: 5EAT) using the homology modelling program in the molecular operation environment (Molecular Operating Environment (MOE), 2015.10; Chemical Computing Group Inc., 1010 Sherbooke St. West, Suite #910, Montreal, QC, Canada, H3A 2R7, 2015).

### Transcript abundance analysis of *GmAFS* using quantitative reverse‐transcription PCR

The expression of *GmAFS* in the two genetically related breeding lines: TN02‐226 and TN02‐275, was analysed in their roots with or without the treatment with SCN HG type 1.2.5.7 (race 2) (Mazarei *et al*., 2011). The expression of *GmAFS* in the soybean Williams 82 was analysed in leaves of the control plants, the plants treated with *T. urticae* and the plants treated with methyl jasmonate. Gene expression was measured using quantitative reverse‐transcription PCR (qRT‐PCR) as previously reported (Lin *et al*., 2013). When performing quantitative RT‐PCR analysis for *GmAFS*, soybean ubiquitin‐3 gene (*GmUBI‐3*, GenBank accession D28123) was used as a reference gene. The sequences of gene specific primers were as follows: GmAFS‐rt‐F 5′‐GCTTGGATTTCATCTTCGGGA‐3′, GmAFS‐rt‐R 5′‐GGTCCCTAAATCATTGCACAATCT‐3′, GmUBI‐3‐F 5′‐GTGTAATGTTGGATGTGTTCCC‐3′, and GmUBI‐3‐R 5′‐ACACAATTGAGTTCAACACAAACCG‐3′. All qRT‐PCR assays were conducted in triplicate. PCR efficiencies for target and reference genes were equal among samples. Ct values and relative abundance were calculated using software supplied with the Applied Biosystems 7900 HT Fast Real‐Time PCR system. The qRT‐PCR data were analysed as previously described by Yuan *et al*. (2006).

### Construction of binary vectors for root transformation and generation of transgenic soybean hairy roots

The pCAMBIA 1305.2 vector was used as a backbone binary vector. Our construct was built based on previously described plasmids pJL‐OFP and pJL‐OFP‐35S:GUS (Lin *et al*., 2013), which contained the coding sequence of an orange fluorescent protein (OFP) reporter gene, originally called as *pporRFP* (Mann *et al*., 2012). The *GmAFS* cDNA was inserted into the BamHI and SacI sites of pJL‐OFP‐35S:GUS to replace the GUS gene, which resulted in the pJL‐OFP‐35S:GmAFS construct. The constructs including pJL‐OFP and pJL‐OFP‐35S:GmAFS were introduced into the *Agrabacterium rhizogenes* strain K599 by the freeze‐thaw method (Chen *et al*., 1994). To test GmAFS's role in soybean cyst nematode resistance, transgenic soybean hairy root with overexpression of *GmAFS* using Williams 82 soybean was generated as previously described (Lin *et al*., 2013). After about 4 weeks, the hairy roots grew to approximately 10 cm in length. Transgenic soybean roots were screened using dual fluorescent protein flashlight (NightSea, Lexington, MA) to detect *OFP* expression. The tap roots and hairy roots without *OFP* expression were excised off from the composite plant. The tap root and OFP‐negative hairy roots and all but one transgenic hairy root were excised under the wounding site of the soybean composite plants containing OFP‐positive transgenic hairy roots harbouring pJL‐OFP or pJL‐OFP‐35S:GmAFS. The composite soybean plants with a single transgenic hairy root were subjected to SCN bioassays.

### SCN treatment for breeding lines and SCN bioassay on transgenic hairy root overexpressing *GmAFS*

The cultures of SCN HG type 1.2.5.7 (race 2) and SCN HG type 0 (race 3) were maintained in Dr. Arelli's laboratory (Arelli *et al*., 2000). SCN HG type 1.2.5.7 (race 2) was used in 6‐day treatment on the soybean breeding lines, TN02‐226 (SCN resistant) and TN02‐275 (SCN susceptible) (Mazarei *et al*., 2011). SCN HG type 0 (race 3) was used for in 35‐day bioassay on transgenic hairy root overexpressing *GmAFS*. Active second‐stage juvenile (J2) nematodes (1000 J2 SCN/plant) were used for inoculation. SCN inoculation and bioassay for transgenic hairy root overexpressing *GmAFS* were performed as previously described (Lin *et al*., 2016). The inoculation was carried out in a growth chamber for 2 days with 16‐h day length and 25 °C day/night temperature. The composite soybean plants with inoculated hairy roots were transplanted to sterile sand in 50‐cm^3^ cone‐tainers (12 cm in length, 2.5 cm inside diameter) randomly arranged within the tray (Stuewe and Sons, Tangent, OR) and maintained for 35 days in the growth chamber with 16‐h day length and 22 °C day/night temperature and 100–110 μmol/cm/s light intensity. The composite plants were watered every other day and fertilized weekly with Peters Professional fertilizer (Scotts, Marysville, OH). The result was combination of four independent experiments with four to five plants analysed in each bioassay. Female index, a well‐known method to compare the soybean resistance level (Niblack, 2005), was used in this study. Female index was calculated as average number of adult females and cysts for the transgenic soybean divided by average number of females and cysts for control line, multiplied by 100. The susceptibility of hairy root harbouring pJL‐*OFP*‐35S:*GmAFS* at the cyst stage was calculated based on female index. Each of the SCN bioassays was conducted with four replicates.

### Plant volatile collection and identification

Volatiles emitted from the *T. urticae*‐treated and control soybean plants were collected in an open headspace sampling system (Analytical Research Systems, Gainesville, FL). Three plants grown in a pot with root systems wrapped with aluminium foil were placed in a glass chamber (30 cm high × 10 cm diameter), with a removable O‐ring snap lid with an air outlet port. The charcoal‐purified air was passed into the chamber at a flow rate of 0.8 L min^−1^ from the top through a Teflon^®^ hose. Volatiles were collected by pumping air from the chamber through a SuperQ volatile collection trap (Analytical Research Systems, Gainesville, FL). After 16‐h collection, 100 μL of methylene chloride containing 1‐octanol (0.003%) as an internal standard was used to elute the volatiles into a glass tube for quantification. The volatile analysis was performed in triplicate to confirm the volatile products.

Plant volatiles and volatile terpenoids from TPS enzyme assays were analysed by a Shimadzu 17A gas chromatograph coupled to a Shimadzu QP5050A quadrupole mass selective detector. Compounds' separation was performed on a Restek SHR5XLB column with 30 m × 0.25 mm internal diameter × 0.25 μm thickness (Shimadzu, Columbia, MD). Helium was used as the carrier gas at flow rate of 1.7 mL min^−1^, and a splitless injection (injection temperature 250 °C) was used. A temperature gradient of 5 °C min^−1^ from 60 °C (6 min hold) to 300 °C was applied. Products were identified based on the National Institute of Standards and Technology (NIST) mass spectral database by comparing of retention times and mass spectra with authentic reference compounds if available. Compound quantification was performed as previously reported (Chen *et al*., 2009). Representative single‐ion peaks of each compound were integrated and compared with the equivalent response of the internal standard (single‐ion method).

### Statistical analysis

Statistical analysis for gene expression and female index was tested with a one‐way ANOVA followed by Fisher's LSD with an alpha level of 0.05 using R software (version 3.1.0) (R Foundation for Statistical Computing, Vienna, Austria).

## Conflict of interest

The authors declare that they have no competing interests.

## References

[pbi12649-bib-0001] Arelli, P.R. , Sleper, D.A. , Yue, P. and Wilcox, J.A. (2000) Soybean reaction to Races 1 and 2 of *Heterodera glycines* . Crop Sci. 40, 824–826.

[pbi12649-bib-0002] Bennett, R.N. and Wallsgrove, R.M. (1994) Secondary metabolites in plant defense mechanisms. New Phytol. 127, 617–633. 10.1111/j.1469-8137.1994.tb02968.x 33874382

[pbi12649-bib-0003] Boeve, J.L. , Lengwiler, U. , Dorn, S. , Turlings, T.C.J. and Tollsten, L. (1996) Volatiles emitted by apple fruitlets infested by larvae of the European apple sawfly. Phytochem. Lett. 42, 373–381.

[pbi12649-bib-0004] Chen, H. , Nelson, R.S. and Sherwood, J.L. (1994) Enhanced recovery of transformants of *Agrobacterium‐tumefaciens* after freeze‐thaw transformation and drug selection. Biotechniques, 16, 664–670. 8024787

[pbi12649-bib-0005] Chen, F. , Tholl, D. , D'Auria, J.C. , Farooq, A. , Pichersky, E. and Gershenzon, J. (2003) Biosynthesis and emission of terpenoid volatiles from *Arabidopsis* flowers. Plant Cell, 15, 481–494. 12566586 10.1105/tpc.007989 PMC141215

[pbi12649-bib-0006] Chen, F. , Al‐Ahmad, H. , Joyce, B. , Zhao, N. , Köllner, T.G. , Degenhardt, J. and Stewart, C.N. (2009) Within‐plant distribution and emission of sesquiterpenes from *Copaifera officinalis* . Plant Physiol. Biochem. 47, 1017–1023. 19648019 10.1016/j.plaphy.2009.07.005

[pbi12649-bib-0007] Chen, F. , Tholl, D. , Bohlmann, J. and Pichersky, E. (2011) The family of terpene synthases in plants: a mid‐size family of genes for specialized metabolism that is highly diversified throughout the kingdom. Plant J. 66, 212–229. 21443633 10.1111/j.1365-313X.2011.04520.x

[pbi12649-bib-0008] Dai, H.Y. , Meyer, M. , Stepaniants, S. , Ziman, M. and Stoughton, R. (2002) Use of hybridization kinetics for differentiating specific from non‐specific binding to oligonucleotide microarrays. Nucleic Acids Res. 30, e86. 12177314 10.1093/nar/gnf085 PMC134259

[pbi12649-bib-0009] Danner, H. , Boeckler, G.A. , Irmisch, S. , Yuan, J.S. , Chen, F. , Gershenzon, J. , Unsicker, S.B. *et al* (2011) Four terpene synthases produce major compounds of the gypsy moth feeding‐induced volatile blend of *Populus trichocarpa* . Phytochemistry, 72, 897–908. 21492885 10.1016/j.phytochem.2011.03.014

[pbi12649-bib-0010] Degenhardt, J. , Köllner, T. and Gershenzon, J. (2009) Monoterpene and sesquiterpene synthases and the origin of terpene skeletal diversity in plants. Phytochemistry, 70, 1621–1637. 19793600 10.1016/j.phytochem.2009.07.030

[pbi12649-bib-0011] Du, Y.J. , Poppy, G.M. , Powell, W. , Pickett, J.A. , Wadhams, L.J. and Woodcock, C.M. (1998) Identification of semiochemicals released during aphid feeding that attract parasitoid *Aphidius ervi* . J. Chem. Ecol. 24, 1355–1368.

[pbi12649-bib-0012] Facchini, P.J. and Chappell, J. (1992) Gene family for an elicitor‐induced sesquiterpene cyclase in tobacco. Proc. Natl Acad. Sci. USA, 89, 11088–11092. 1438319 10.1073/pnas.89.22.11088 PMC50489

[pbi12649-bib-0013] Fäldt, J. , Martin, D. , Miller, B. , Rawat, S. and Bohlmann, J. (2003) Traumatic resin defense in Norway spruce (*Picea abies*): methyl jasmonate‐induced terpene synthase gene expression, and cDNA cloning and functional characterization of (+)‐3‐carene synthase. Plant Mol. Biol. 51, 119–133. 12602896 10.1023/a:1020714403780

[pbi12649-bib-0014] Gershenzon, J. and Dudareva, N. (2007) The function of terpene natural products in the natural world. Nat. Chem. Biol. 3, 408–414. 17576428 10.1038/nchembio.2007.5

[pbi12649-bib-0015] Green, S. , Friel, E.N. , Matich, A. , Beuning, L.L. , Cooney, J.M. , Rowan, D.D. and MacRae, E. (2007) Unusual features of a recombinant apple alpha‐farnesene synthase. Phytochemistry, 68, 176–188. 17140613 10.1016/j.phytochem.2006.10.017

[pbi12649-bib-0016] Green, S. , Squire, C.J. , Nieuwenhuizen, N.J. , Baker, E.N. and Laing, W. (2009) Defining the potassium binding region in an apple terpene synthase. J. Biol. Chem. 284, 8661–8669. 19181671 10.1074/jbc.M807140200 PMC2659225

[pbi12649-bib-0018] Hartman, G.L. , West, E.D. and Herman, T.K. (2011) Crops that feed the World 2. Soybean‐worldwide production, use, and constraints caused by pathogens and pests. Food Sec. 3, 5–17.

[pbi12649-bib-0019] Huang, M.S. , Abel, C. , Sohrabi, R. , Petri, J. , Haupt, I. , Cosimano, J. , Gershenzon, J. *et al* (2010) Variation of herbivore‐induced volatile terpenes among *Arabidopsis* ecotypes depends on allelic differences and subcellular targeting of two terpene synthases, TPS02 and TPS03. Plant Physiol. 153, 1293–1310. 20463089 10.1104/pp.110.154864 PMC2899926

[pbi12649-bib-0020] James, D. (2005) Further field evaluation of synthetic herbivore‐induced plant volatiles as attractants for beneficial insects. J. Chem. Ecol. 31, 481–495. 15898496 10.1007/s10886-005-2020-y

[pbi12649-bib-0021] Koenning, S.R. and Wrather, J.A. (2010) Suppression of soybean yield potential in the continental United States from plant diseases estimated from 2006 to 2009. Plant Health Prog.

[pbi12649-bib-0022] Larkin, M.A. , Blackshields, G. , Brown, N.P. , Chenna, R. , McGettigan, P.A. , McWilliam, H. , Valentin, F. *et al* (2007) Clustal W and Clustal X version 2.0. Bioinformatics, 23, 2947–2948. 17846036 10.1093/bioinformatics/btm404

[pbi12649-bib-0023] Lin, J. , Mazarei, M. , Zhao, N. , Zhu, J.W.J. , Zhuang, X.F. , Liu, W.S. , Pantalone, V.R. *et al* (2013) Overexpression of a soybean salicylic acid methyltransferase gene confers resistance to soybean cyst nematode. Plant Biotechnol. J. 11, 1135–1145. 24034273 10.1111/pbi.12108

[pbi12649-bib-0024] Lin, J. , Mazarei, M. , Zhao, N. , Hatcher, C.N. , Wuddineh, W.A. , Rudis, M. , Tschaplinski, T.J. *et al* (2016) Transgenic soybean overexpressing GmSAMT1 exhibits resistance to multiple‐HG types of soybean cyst nematode *Heterodera glycines* . Plant Biotechnol. J. 14, 2100–2109. 27064027 10.1111/pbi.12566 PMC5095773

[pbi12649-bib-0025] Liu, J. , Huang, F. , Wang, X. , Zhang, M. , Zheng, R. , Wang, J. and Yu, D. (2014) Genome‐wide analysis of terpene synthases in soybean: functional characterization of GmTPS3. Gene, 544, 83–92. 24768723 10.1016/j.gene.2014.04.046

[pbi12649-bib-0026] Mallinger, R.E. , Hogg, D.B. and Gratton, C. (2011) Methyl salicylate attracts natural enemies and reduces populations of soybean aphids (Hemiptera: Aphididae) in soybean agroecosystems. J. Econ. Entomol. 104, 115–124. 21404848 10.1603/ec10253

[pbi12649-bib-0027] Mann, D.G.J. , LaFayette, P.R. , Abercrombie, L.L. , King, Z.R. , Mazarei, M. , Halter, M.C. , Poovaiah, C.R. *et al* (2012) Gateway‐compatible vectors for high‐throughput gene functional analysis in switchgrass (*Panicum virgatum* L.) and other monocot species. Plant Biotechnol. J. 10, 226–236. 21955653 10.1111/j.1467-7652.2011.00658.x

[pbi12649-bib-0028] Mazarei, M. , Liu, W. , Al‐Ahmad, H. , Arelli, P.R. , Pantalone, V.R. and Stewart, C.N. (2011) Gene expression profiling of resistant and susceptible soybean lines infected with soybean cyst nematode. Theor. Appl. Genet. 123, 1193–1206. 21800143 10.1007/s00122-011-1659-8

[pbi12649-bib-0029] Moraes, M.C.B. , Laumann, R. , Sujii, E.R. , Pires, C. and Borges, M. (2005) Induced volatiles in soybean and pigeon pea plants artificially infested with the neotropical brown stink bug, *Euschistus heros*, and their effect on the egg parasitoid, *Telenomus podisi* . Entomol. Exp. Appl. 115, 227–237.

[pbi12649-bib-0030] Niblack, T.L. (2005) Soybean cyst nematode management reconsidered. Plant Dis. 89, 1020–1026. 10.1094/PD-89-1020 30791267

[pbi12649-bib-0031] Ntalli, N.G. , Ferrari, F. , Giannakou, I. and Menkissoglu‐Spiroudi, U. (2010) Phytochemistry and nematicidal activity of the essential oils from 8 Greek Lamiaceae aromatic plants and 13 terpene components. J. Agric. Food Chem. 58, 7856–7863. 20527965 10.1021/jf100797m

[pbi12649-bib-0032] Oerke, E.C. (2006) Crop losses to pests. J. Agric. Sci. 144, 31–43.

[pbi12649-bib-0033] Oka, Y. , Nacar, S. , Putievsky, E. , Ravid, U. , Yaniv, Z. and Spiegel, Y. (2000) Nematicidal activity of essential oils and their components against the root‐knot nematode. Phytopathology, 90, 710–715. 18944489 10.1094/PHYTO.2000.90.7.710

[pbi12649-bib-0034] Phillips, M.A. , Wildung, M.R. , Williams, D.C. , Hyatt, D.C. and Croteau, R. (2003) cDNA isolation, functional expression, and characterization of (+)‐α‐pinene synthase and (−)‐α‐pinene synthase from loblolly pine (*Pinus taeda*): Stereocontrol in pinene biosynthesis. Arch. Biochem. Biophys. 411, 267–276. 12623076 10.1016/s0003-9861(02)00746-4

[pbi12649-bib-0035] Rasmann, S. , Köllner, T.G. , Degenhardt, J. , Hiltpold, I. , Toepfer, S. , Kuhlmann, U. , Gershenzon, J. *et al* (2005) Recruitment of entomopathogenic nematodes by insect‐damaged maize roots. Nature, 434, 732–737. 15815622 10.1038/nature03451

[pbi12649-bib-0036] Schmutz, J. , Cannon, S.B. , Schlueter, J. , Ma, J. , Mitros, T. , Nelson, W. , Hyten, D.L. *et al* (2010) Genome sequence of the palaeopolyploid soybean. Nature, 463, 178–183. 20075913 10.1038/nature08670

[pbi12649-bib-0037] Scutareanu, P. , Bruin, J. , Posthumus, M.A. and Drukker, B. (2003) Constitutive and herbivore‐induced volatiles in pear, alder and hawthorn trees. Chemoecology, 13, 63–74.

[pbi12649-bib-0038] Shrivastava, G. , Rogers, M. , Wszelaki, A. , Panthee, D.R. and Chen, F. (2010) Plant volatiles‐based insect pest management in organic farming. Crit. Rev. Plant Sci. 29, 123–133.

[pbi12649-bib-0039] Tamura, K. , Stecher, G. , Peterson, D. , Filipski, A. and Kumar, S. (2013) MEGA6: molecular evolutionary genetics analysis version 6.0. Mol. Biol. Evol. 30, 2725–2729. 24132122 10.1093/molbev/mst197 PMC3840312

[pbi12649-bib-0040] War, A.R. , Paulraj, M.G. , Ahmad, T. , Buhroo, A.A. , Hussain, B. , Ignacimuthu, S. and Sharma, H.C. (2012) Mechanisms of plant defense against insect herbivores. Plant Signal Behav. 7, 1306–1320. 22895106 10.4161/psb.21663 PMC3493419

[pbi12649-bib-0041] Whittington, D.A. , Wise, M.L. , Urbansky, M. , Coates, R.M. , Croteau, R.B. and Christianson, D.W. (2002) Bornyl diphosphate synthase: structure and strategy for carbocation manipulation by a terpenoid cyclase. Proc. Natl Acad. Sci. USA, 99, 15375–15380. 12432096 10.1073/pnas.232591099 PMC137724

[pbi12649-bib-0042] Yuan, J.S. , Reed, A. , Chen, F. and Stewart, C.N. (2006) Statistical analysis of real‐time PCR data. BMC Bioinformatics, 7, 85. 16504059 10.1186/1471-2105-7-85 PMC1395339

[pbi12649-bib-0043] Yuan, J.S. , Köllner, T.G. , Wiggins, G. , Grant, J. , Degenhardt, J. and Chen, F. (2008) Molecular and genomic basis of volatile‐mediated indirect defense against insects in rice. Plant J. 55, 491–503. 18433439 10.1111/j.1365-313X.2008.03524.x

[pbi12649-bib-0044] Zhao, N. , Wang, G.‐D. , Norris, A. , Chen, X.‐L. and Chen, F. (2013) Studying plant secondary metabolism in the age of genomics. Crit. Rev. Plant Sci. 32, 369–382.

[pbi12649-bib-0045] Zhu, J.W. , Cosse, A.A. , Obrycki, J.J. , Boo, K.S. and Baker, T.C. (1999) Olfactory reactions of the twelve‐spotted lady beetle, *Coleomegilla maculata* and the green lacewing, *Chrysoperla carnea* to semiochemicals released from their prey and host plant: electroantennogram and behavioral responses. J. Chem. Ecol. 25, 1163–1177.

[pbi12649-bib-0046] Zhuang, X. , Köllner, T.G. , Zhao, N. , Li, G. , Jiang, Y. , Zhu, L. , Ma, J. *et al* (2012) Dynamic evolution of herbivore‐induced sesquiterpene biosynthesis in sorghum and related grass crops. Plant J. 69, 70–80. 21880075 10.1111/j.1365-313X.2011.04771.x

